# Improving implementation of evidence‐based therapies for heart failure

**DOI:** 10.1002/clc.23845

**Published:** 2022-07-05

**Authors:** Adam D. DeVore, Hayden B. Bosworth, Bradi B. Granger

**Affiliations:** ^1^ Duke Clinical Research Institute, Duke University School of Medicine Durham North Carolina USA; ^2^ Department of Medicine Duke University School of Medicine Durham North Carolina USA; ^3^ Center of Innovation to Accelerate Discovery and Practice Transformation Durham Veterans Affairs Medical Center Durham North Carolina USA; ^4^ Department of Population Health Sciences Duke University Medical Center Durham North Carolina USA; ^5^ Department of Medicine Division of General Internal Medicine, Duke University Medical Center Durham North Carolina USA; ^6^ Department of Psychiatry and Behavioral Sciences Duke University Medical Center Durham North Carolina USA; ^7^ Duke University School of Nursing, Duke University School of Medicine Durham North Carolina USA

**Keywords:** clinical trials, heart failure, implementation science, quality improvement

## Abstract

Treatment options for patients with heart failure have improved rapidly over the last few decades. Data from large scale clinical trials demonstrate that medical and device therapies can improve quality of life, reduce hospitalizations for acute heart failure, and reduce mortality. However, the use of many of these therapies in routine practice is remarkably low. There are many reasons for suboptimal implementation of evidence‐based therapies for heart failure, and we believe addressing the large gap between what can be accomplished in clinical trials versus routine practice is a critical and urgent public health issue. In this review, we outline reasons for this implementation gap and review recent studies attempting to address this issue. We also provide recommendations for future interventions and areas of clinical investigation to improve implementation for patients with heart failure.

## 1 INTRODUCTION

Heart failure (HF) affects over 6 million people in the United States alone.^1^ Patients with HF have a high burden of symptoms, require medications, and requisite lifestyle changes, and are at increased risk of poor outcomes, including subsequent HF hospitalization and death.^2^ Poor outcomes have persisted over time despite the introduction of new therapies for HF, including medications, implantable technologies, and more flexible home‐based approaches to support shared decision‐making, goal‐setting, and rehabilitation care.

Medications for HF target multiple maladaptive responses in HF including neurohormonal overactivation and led to improved clinical outcomes in large‐scale clinical trials.^3^, ^4^, ^5^, ^6^, ^7^, ^8^ However, the use of these medications remains low in in clinical practice, and is a paradigm case of suboptimal implementation of evidence‐based therapies for HF. For example, for patients with chronic HF with reduced ejection fraction (HFrEF), the use of angiotensin receptor‐neprilysin inhibitors (ARNI) reduced the risk of death by 16% compared with the previous standard of care, angiotensin‐converting enzyme inhibitors (ACEI) (hazard ratio [HR]: 0.84; 95% confidence interval [CI]: 0.76−0.93) in the PARADIGM‐HF trial.^9^ However, two large studies of US‐based registries from 2015 to 2017 found that <15% of patients potentially eligible for ARNI therapy were prescribed it.^10^, ^11^ There are many reasons that improvements observed in clinical trials have not been seen in routine practice, many of these are outlined below. Addressing the large gap between what can be accomplished in clinical trials and routine practice is a critical and urgent public health issue.

### 1.1 Potential reasons for a HF implementation gap

The implementation gap between what is possible in clinical trials versus routine practice is not unique to HF. Over two decades ago, the Institute of Medicine published Crossing the Quality Chasm: A New Health System for the 21st Century.^12^ This report highlighted that the addition of new medical knowledge and technology had outpaced the ability of the US healthcare system to (1) translate knowledge into practice and (2) use new technology to improve care and outcomes. Large gaps in adoption of existing evidence were apparent in many aspects of care from prevention, acute care including myocardial infarction, and management of chronic diseases such as diabetes.^13^

**Figure 1:**
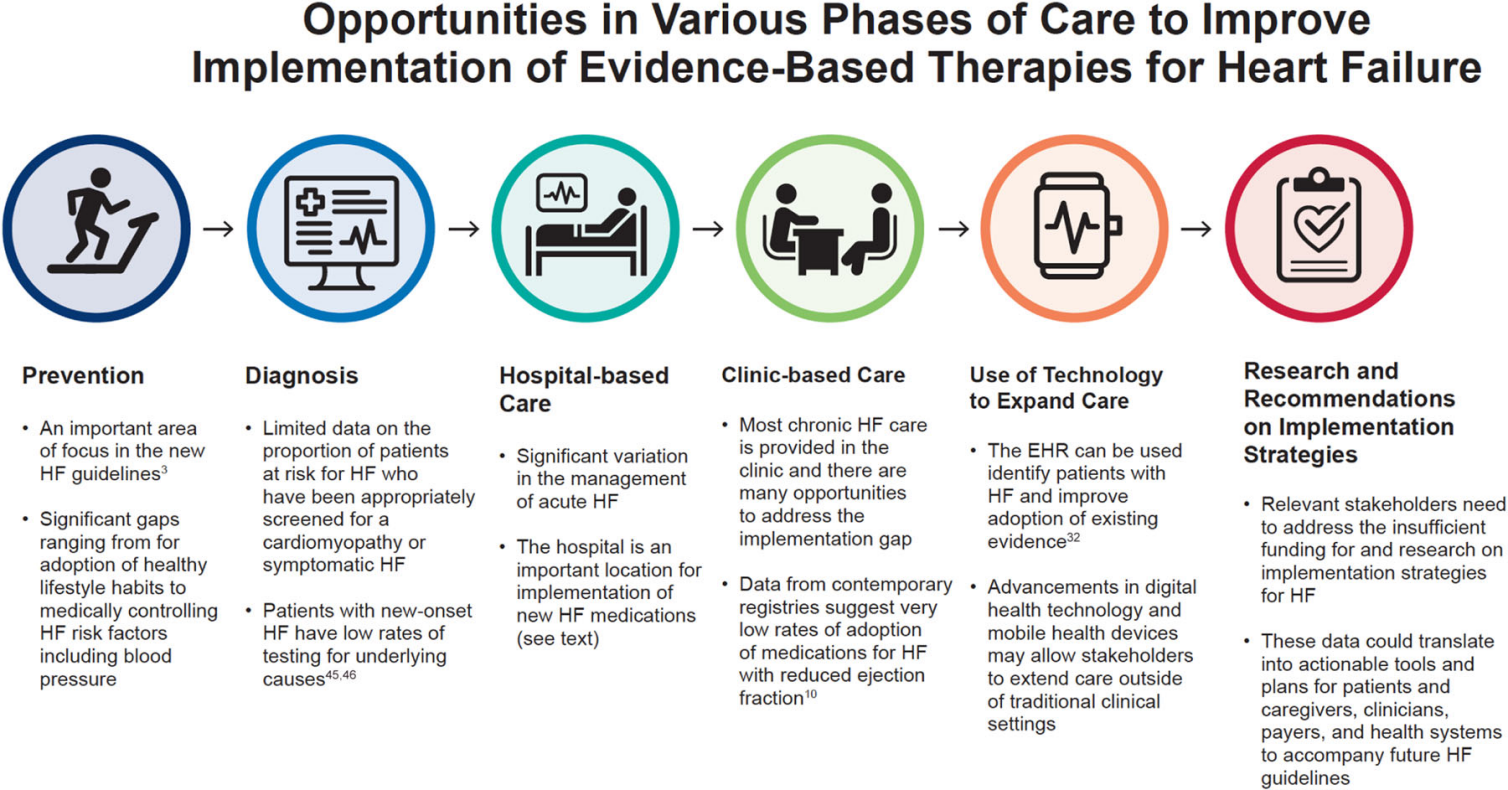
Opportunities in various phases of care to improve implementation of evidence‐based therapies for heart failure (HF). In this figure, we highlight opportunities to improve implementation in multiple aspects of HF care, from prevention of HF to patients with symptomatic heart failure, and across the care continuum.^3^, ^10^, ^32^, ^45^, ^46^

The causes for these gaps in care are similar in chronic HF. In the Table 1, we describe reported reasons for a HF implementation gap. These complex and interrelated drivers affect not only HF but the broader population of patients with chronic cardiovascular disease. Adding to the complexity is that while new knowledge in medicine is rapidly advancing, the issues affecting implementation of that knowledge in clinical practice have changed very little and are not limited to clinician awareness. Rather, the health system itself is fraught with obstacles that impede adoption including poor interoperability of patient monitoring technologies with electronic health records (EHR), lack of communication across care settings, and payment models that fail to fund the cadre of downstream clinicians and family caregivers who make high quality, long‐term care possible. In Figure 1 we highlight that the disconnect between *knowing* and *doing* can be observed as inaction across multiple phases of HF care and across the HF care continuum encompassing patients, clinicians, payers, and policy‐makers.

**Table 1:** Potential reasons a heart failure implementation gap.

Reason	Description
Clinician knowledge gap	This is an important factor, especially given the pace of new knowledge and technology, though only partly explains the HF implementation gap.^12^ Interventions on education can be effective at improving processes of care and select patient outcomes though there are limited data on best practices for education interventions to patients or clinicians in HF.^29^, ^41^
Patient and caregiver awareness	It is unclear how much limited patient and caregiver awareness of HF and its prognosis impacts the implementation gap.^42^ In the EPIC‐HF study, an electronically delivered patient activation intervention was able to increase use of evidence‐based therapies for HFrEF.^33^ This suggests interventions targeting patients awareness are an opportunity for improving HF care.
	
Trial participants are not representative of clinical practice	Clinical trials are intentionally designed to evaluate safety and efficacy of an intervention in a narrow population. As such, clinical trial participants are different than patients with HF in routine practice and registries.^43^ While these differences are important, they are unlikely to explain the large implementation gap observed given that a large proportion of patients with HF still fit basic trial eligibility criteria for HF therapies.^44^
Patient costs, for example, prescription copayments	This is commonly encountered in routine practice, especially in the United States, and is likely an important barrier for newer therapies with higher copayments. However, low rates of less expensive generic medications persist, such as mineralocorticoid receptor antagonists.^16^
Limited evidence on implementation strategies	We feel this is an important barrier to improving HF care and findings on implementation strategies are likely to have implications for improving other areas of medical care.

Abbreviations: EPIC‐HF, Electronically Delivered, Patient‐Activation Tool for Intensification of Medications for Chronic HFrEF; HF, heart failure; HFrEF, HF with reduced ejection fraction.

### 1.2 Strategies to improve implementation in HF

**Figure 2:**
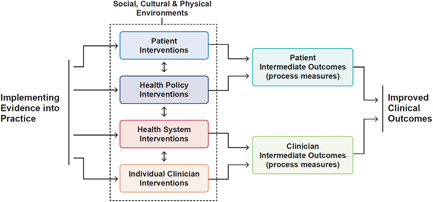
Framework for improving implementation across the care continuum. In this figure, we display a framework that highlights various levels where implementation strategies can be initiated impacting patients, clinicians, payers, and policy‐makers. Adapted from Figure 1 in Chan et al.^23^

In Figure 2 we propose a framework for addressing a HF implementation gap based on prior work.^14^ Given that this implementation gap is influenced by multiple stakeholders in care delivery including patients, clinicians, caregivers, payers, and so forth, we propose solutions that are multilevel and subsequently can influence behavior through care delivery pathways. We include multiple suggestions specifically for clinicians as they play an essential role reducing this implementation gap. These suggestions have not all been evaluated in the context of HF, and a greater focus on prospective evaluation of implementation strategies in HF is needed.

### 1.3 Meet patients where they are (e.g., in‐hospital initiation)

Hospitalizations for acute HF are a sentinel event for patients living with HF in that they are both disruptive for patients and families, and they are a marker of increased risk for poor future outcomes, including death.^15^ This water‐shed event combined with the fact that in‐hospital initiation of HFrEF medications affords a convenient opportunity for monitoring of blood pressure, renal function, and so forth, makes the hospital an important location for implementation of new HF medications and other therapies. In separate analyses from the Get With The Guidelines‐HF quality improvement program, patients with HFrEF hospitalized for acute HF, and eligible for ARNIs and mineralocorticoid receptor antagonists were followed for 1 year after discharge.^16^ When initiation of these therapies was deferred until after hospital discharge, it was observed that >75% of the time these therapies were not initiated over the next year. In contrast, when patients did initiate therapy in the hospital >80% of patients were still prescribed the therapy 12 months later (though the absolute number of in‐hospital initiations was low).

Multiple clinical trials support a strategy of in‐hospital initiation of evidence‐based therapies for HFrEF. In the Initiation Management Predischarge: Process for Assessment of Carvedilol Therapy in Heart Failure (IMPACT‐HF) trial, participants with HFrEF were randomized to prehospital discharge initiation of an evidence‐based beta‐blocker versus usual care.^17^ At 60 days after randomization, participants randomized to the predischarge initiation strategy had significantly higher rates of use of beta‐blocker compared with usual care (91% vs. 73%, *p* < .0001), and there was no difference in hospital length of stay or rates of serious adverse events in the two groups. In the Prospective Comparison of ARNI with ACEI to Determine Impact on Global Mortality and Morbidity in HF (PIONEER‐HF) trial, participants with HFrEF hospitalized with acute HF were randomized to a strategy of in‐hospital initiation of ARNI versus enalapril.^18^ The strategy of in‐hospital initiation of ARNI therapy, compared with enalapril, led to both a greater reduction in N‐terminal pro‐B‐type natriuretic peptide concentration from baseline and improved clinical outcomes at 4−8 weeks after randomization.^19^ Key safety outcomes including worsening renal function, hyperkalemia, symptomatic hypotension, and angioedema, were also similar in both arms.^18^ Recent data also suggest the initiation of sodium–glucose cotransporter 2 (SGLT2) inhibitors around the time of hospitalization for acute HF is safe and effective. In the Effect of Sotagliflozin on Cardiovascular Events in Patients with Type 2 Diabetes Post Worsening HF (SOLOIST‐WHF) trial, the combined SGLT 1 and 2 inhibitor, sotagliflozin, was compared to placebo in patients with type 2 diabetes and a recent worsening HF event across a range of left ventricular ejection fractions.^20^ Patients were initiated on therapy either before hospital discharge or within 3 days of discharge, and sotagliflozin use was well‐tolerated and led to a lower number of cardiovascular deaths and hospitalizations and urgent visits for HF compared with placebo. Similarly, in the EMPULSE trial, use of the SGLT2 inhibitor empagliflozin was compared to placebo in participants hospitalized with acute HF, regardless of type 2 diabetes or left ventricular ejection fraction.^21^ Participants were enrolled between 24 h and 5 days after admission and empagliflozin use was well‐tolerated and led to an improvement in a composite outcome of clinical events or Kansas City Cardiomyopathy Questionnaire total symptom score. The use of the SGLT2 inhibitor dapagliflozin is also currently being evaluated in the Dapagliflozin and Effect on Cardiovascular Events in Acute HF (DAPA ACT HF) trial.^22^

The hospital is an important target for improving implementation for medical and device therapies for patients with HF. However, there are limitations to focusing primarily on hospital‐based initiation. First, hospitals face pressure to minimize lengths of stay from payers and patients and families. Second, while hospitalizations are important events for patients with HF, they still represent a small proportion of time that a patient lives with HF. Third, targeting the hospital for implementation focuses only on patients with more advanced disease and misses a large proportion of patients that may benefit from earlier intervention on HF and new‐onset cardiomyopathies.

### 1.4 Provide feedback and education to hospital quality teams and clinicians

A foundational component of influencing clinical care is measuring care and providing feedback on clinical performance. In a prior report of a large implementation science working group, (1) audit and feedback of clinical performance and (2) educational outreach to clinicians were highlighted as effective strategies for improving medical care, including in cardiovascular disease.^23^ This approach is used by multiple large HF quality improvement programs, such as the American Heart Association's Get With The Guildelines‐HF program, that provide feedback and education on HF process measures and outcomes to hospital‐based quality improvement teams.^24^, ^25^ These programs have been successful at focusing large components of the US healthcare system on quality improvement in HF. For example, for hospitals that participate in the Get With The Guidelines‐HF program, assessments of left ventricular function are >99% among hospitalized patients.^26^ This impact on hospital‐based HF care is remarkable though the impact on postdischarge outcomes has been modest.^27^ The reasons for the limited impact on postdischarge outcomes are unknown but may be related to the fragmented nature of HF care delivery in the United States. That is, many patients with HF are lacking longitudinal, continuity of care. There are often different clinicians treating patients in the hospital versus clinic, and communication across care settings remains a challenge despite the use of EHR.

Our group helped lead two large, cluster‐randomized trials evaluating different strategies related to hospital‐based feedback and education. In one, 147 US hospitals already participating in the Get With The Guidelines‐HF program were randomized to the usual Get With The Guidelines‐HF quality improvement program versus additional personalized quality improvement reports paired with tailored teleconferences, webinars, and specialized tool kits on HF care.^28^ These additional components represented additional audit and feedback of hospital‐based HF clinical performance and HF education to hospital‐based clinical teams. The primary outcome was improvement in a hospital‐level composite quality of care score. From 2009 to 2010, 73 hospitals (33 886 patients) received the intervention, and 74 hospitals (37 943 patients) did not. One year after the intervention, both the intervention and control arms had a similar mean change in percentage points in their composite quality score (intervention arm: absolute change, +0.31 [standard error, 1.51] vs. control arm: +3.18 [standard error, 1.68]; *p* = .21).

In another large trial, Care Optimization Through Patient and Hospital Engagement Clinical Trial for HF (CONNECT‐HF), 161 US hospitals were randomized to usual care versus an intervention that focused on multiple domains for HF care: hospital discharge care, transitions of care, and outpatient care delivery.^29^ This intervention also centered around audit and feedback and HF education, but it was more intensive than the prior study and included a focus on HF care outside of the hospital and targeted both inpatient and outpatient teams. From 2017 to 2020, 82 hospitals (2675 patients) received the intervention and 79 hospitals (2972 patients) received usual care. The coprimary outcomes were a composite of first HF rehospitalization or all‐cause mortality and change in an opportunity‐based composite score for HF quality. HF rehospitalization or death occurred in 38.6% in the intervention group versus 39.2% in usual care (adjusted HR, 0.92 [95% CI: 0.81−1.05]). The change in quality‐of‐care score was +2.3% in the intervention group versus −1.0% in the usual care group (difference, 3.3% [95% CI: −0.8% to 7.3%]). There was also no significant difference between the two groups in the odds of achieving a higher composite quality score at last follow‐up (adjusted odds ratio, 1.06 [95% CI: 0.93−1.21]).^30^

When examining the experience of feedback and education in HF quality registries and the data in these two large trials, it seems that specific hospital‐based quality improvement programs, such as Get With The Guidelines‐HF, can improve hospital‐based HF care through audit and feedback paired with education. However, as shown in the additional trials, there is likely a ceiling effect to this approach or this approach is very sensitive to the manner in which feedback is administered (i.e., on what process measures and to whom). In addition, as highlighted in CONNECT‐HF, centering HF quality improvement programs in the hospital may not have the necessary impact on outpatient care.

### 1.5 Build better systems that target prescribers

Prescribing HF medical therapy is primarily a clinician‐led behavior and interventions that directly influence prescribing behavior are likely to lead to the largest impact for improving implementation of HF medications. One challenge of initiation and titration of evidence‐based therapies for HF is that medication changes often occur in a healthcare encounter, either during a hospitalization for HF care or a clinic visit. Implementation of evidence‐based therapies for HF may improve with medication optimization programs that consider the entire patient experience, including at home, and do not rely on traditional clinical encounters. For example, a medication optimization program managed by patient navigators, nurses, pharmacists, and a HF specialist was able to remotely increase the use and dose of HF medications for patients with HFrEF at a single center using telephone calls, a home blood pressure monitor, and local laboratory studies.^31^ After identifying 1131 patients through a screen in the EHR, 218 (19%) agreed to participate and 831 (73%) were excluded by their physician. After 3 months, patients in the program noted increases in the use of ACEI/angiotensin II receptor blockers (ARB)/ARNIs (baseline use: 70%, follow‐up use: 86%; *p* < .001) and beta‐blockers (baseline use: 77%, follow‐up use: 92%; *p* < .001) compared with minimal changes in the group excluded by their physician. These findings are encouraging that a remote HF medication titration clinic may be a solution to the current system that uses hospitalizations and clinic visits every 3−6 months to initiate and titrate HF medications though the safety and efficacy of this approach should be further studied in a randomized study. The high rates of exclusion by treating physicians (73%) also raise concern for generalizability of this approach though the reasons for exclusion are unknown.

Nudges are another set of interventions that can target prescribing behavior. Nudges are intended to be effective but not overly intrusive interventions that can lead to clinician behavior change yet still preserve individual choice for treatment recommendations. Potential examples of nudges in prescribing behavior are clinician‐level feedback on prescribing adherence to evidence‐based HF care (either privately to clinicians or publicly with benchmarks to peers), providing monetary incentives for improving prescribing of evidence‐based HF medications, and changing order sets for HF patients to default prescribing of specific medications. As has been previously described, these nudges can exist on a continuum from lower levels of restriction on clinician autonomy (e.g., provide feedback on performance) to higher levels of restriction (e.g., default orders in an admission order set).^14^

A successful example of nudges in HF prescribing was demonstrated the PROMPT‐HF randomized trial.^32^ Clinicians treating patients with HFrEF in the Yale‐New Haven Health System were randomized to an intervention that included a best practice alert embedded in the EHR that guided clinicians to initiate evidence‐based therapies for HFrEF. The alert included information such as left ventricular ejection fraction, recent kidney function, current medications for HFrEF, and opportunities for prescribing new classes of HF therapies. The alert also included a direct link to an order set. The primary outcome was the proportion of patients with HFrEF who had an increase in the number of prescribed classes of medications for HFrEF at 30 days after randomization. The primary outcome occurred in 26% (176/685 participants) in the intervention group versus 19% (117/625) in the usual care group (adjusted risk ratio: 1.41; 95% CI: 1.03−1.93; *p* = .03). This study shows the potential impact of making small changes in the prescribing options for HF clinicians and that interventions in the EHR are likely scalable across large health systems, and that reducing clinical and therapeutic inertia can be impactful. What is not apparent from PROMPT‐HF is the durability of these changes over time as clinicians can fatigue to best practice alerts. This intervention is also still anchored to traditional clinical encounters, in this case clinic visits, and is limited by how frequently patients are scheduled and able to present to clinic visits for HF care.

### 1.6 Build better systems that target patients and caregivers

Nudges that target patients and families may also be effective strategies for changing prescribing patterns. In the Electronically Delivered, Patient‐Activation Tool for Intensification of Medications for Chronic HFrEF (EPIC‐HF) study, patients with HFrEF in the University of Colorado Health system were consented and randomized to a patient activation intervention on HF care.^33^ Study participants randomized to the intervention received a 3 min video on HFrEF care and one‐page medication checklist before cardiology clinic visits. Participants were then encouraged to discuss changing at least “one thing” to optimize HFrEF medication management during the visit. The primary outcome was any medication intensification (i.e., new initiation or dose increase) from before the clinic visit to 30 days later. The primary outcome occurred in 49% (71/145 participants) in the intervention group versus 30% (43/145) in the usual care group (risk ratio 1.6, 95% CI: 1.2−2.2; *p* = .001). Most of these medication changes occurred at the qualifying clinic visit and involved dose titrations of evidence‐based beta‐blockers. EPIC‐HF was novel in its approach activating patients. Similar to PROMPT‐HF, while the intervention can be easily scaled across large health systems, the durability is unknown and the intervention is still anchored to traditional clinical encounters.

### 1.7 Embrace technology

Noted above are small examples of using the EHR to identify patients with HF and improve aspects of care. However, the advent of digital health technology and mobile health devices is an opportunity to address gaps in care, especially outside of traditional clinical settings. Mobile health devices can be paired with a variety of traditional monitoring equipment (e.g., blood pressure cuffs) as well as novel wearable devices (e.g., heart rate monitors) to continuously capture data that can identify candidates for HF medications, devices, and other interventions.^34^ For example, in the currently enrolling Artificial Intelligence Mobile Health Trial Of A Digital Platform To Optimize GDMT Using Wearable Sensors (AIM‐POWER) study, participants with HFrEF not yet optimized on medical therapy are randomized 1:1 to usual care versus a cloud‐based, clinical decision support platform (BiovitalsHF) that incorporates data from remote monitoring devices and electronic patient reported outcomes to support medication initiation and titration.^35^ The goal of this study, and other similar ones, is to evaluate if a mobile health solution may be more effective than traditional encounters at rapidly incorporating new evidence into practice with titration and titration of medications outside of clinical encounters. The safety of this approach needs evaluation as does more information on the patient and family experience with remote‐based care. In a traditional fee‐for‐service model, reimbursement to clinicians and health systems for this approach may also inhibit adoption though this may be more feasible in a value‐based care system.

### 1.8 Guideline recommendations for implementation

Professional societies in the United States recently published a new iteration of HF guidelines with recommendations on prevention of HF, management of patients with symptomatic HF, and indications for specific devices.^36^ The guidelines highlight the need for implementation studies and novel dissemination and implementation techniques but contain little information on how to operationalize recommendations. Guideline recommendations or accompanying materials need to continue to provide recommendations based on available evidence, but also need to provide actionable tools and plans for patients and caregivers, clinicians, payers, and health systems. These statements have previously been described as “implementation recommendations.”^37^

A salient example in HF care is cardiac rehabilitation. A comprehensive cardiac rehabilitation program is currently recommended to improve functional capacity, exercise tolerance, and health‐related quality of life in patients with HF.^36^ These programs are available throughout the United States and haves been financially approved for patients with chronic HFrEF by Centers for Medicare and Medicaide Services since 2014. However, analysis of Medicare claims data show very low rates of participation among eligible patients, 4.3% in patients with a recent hospitalization and 2.2% with a recent outpatient vist.^38^ An implementation recommendation for cardiac rehabilitation in patients with HF would accompany the guideline recommendation and highlight this low rate of use and provide mechanisms to assess local barriers to use and potential ways to address these barriers and then measure the impact. Elevating and emphasizing the implementation aspect of guideline recommendations would also foster a culture of guideline adoption by relevant stakeholders.

### 1.9 Improving the evidence‐based

An essential aspect of improving implementation for HF is improving funding for and investigation of implementation strategies, particularly with an eye toward unintended consequences. In the field of HF, a prior controversial policy change underscores the importance of a thorough investigation of implementation strategies before widespread adoption. In 2010, the Hospital Readmission Reduction Program was enacted with an overarching goal of reducing preventable hospital readmissions for specific conditions, including HF. The program eventually began using financial incentives to encourage hospitals to improve overall care, by withholding hospital payments for above‐average risk‐adjusted readmission rates. Nearly all hospitals in the United States were impacted by this policy change and studying the effect is difficult as there are limited ways to develop control groups. Analyses evaluating the financial incentives on HF outcomes have found conflicting results though some suggest a very concerning increase in 30‐day postdischarge mortality after a hospitalization for HF.^39^,^40^ An alternative approach to address HF readmissions would have been to generate an evidence base on best methods to reduce readmissions and then study the impact as hospitals adopt these practices over time. The safety and efficacy of these changes could have been prospectively evaluated in a variety of ways, including cluster‐randomized trials that afford for sufficient control groups.

## 2 CONCLUSIONS

Treatment options for patients with HF have expanded at a remarkable rate over the last decade. A primary challenge for relevant stakeholders is to rapidly implement these innovations in practice; to change the story that began in 2001 by the Institute of Medicine to a new story demonstrating that we *can* change health care to improve patient outcomes. We believe there are multiple opportunities to improve care today including through organized health system quality improvement efforts and emphasizing early initiation of HF medications, especially in the hospital. There is also tremendous opportunity to study new technologies and determine if these can aid in the implementation for other aspects of care, including the outpatient setting. For more substantial improvements in care though, the HF community needs to embrace a culture of implementation and advance the field through clinical investigation.

## CONFLICTS OF INTEREST

The following relationships exist related to this manuscript:

Adam D. DeVore reports research funding through his institution from the American Heart Association, Amgen, AstraZeneca, Bayer, Intracellular Therapies, American Regent, Inc., the NHLBI, Novartis, and PCORI. He also provides consulting services for Amgen, AstraZeneca, Bayer, CareDx, InnaMed, LivaNova, Mardil Medical, Novartis, Procyrion, scPharmaceuticals, Story Health, and Zoll. He has also received nonfinancial support from Abbott for educational activities. Hayden B. Bosworth reports research funding through his institution from BeBetter Therapeutics, Boehinger Inghelheim, Improved Patient Outcomes, NLBI, Novo Nordisk, Otsuka, Sanofi, VA. He also provides consulting services for Abott, Novartis, Sanofi, Vidya, Walmart, and Webmed. He is also on the board of directors of Preventric Diagnostics. The remaining author declares no conflict of interest.

## Data Availability

Not applicable.

## References

[1] Virani SS , Alonso A , Aparicio HJ , et al. Heart disease and stroke statistics‐2021 update: a report from the American Heart Association update. Circulation. 2021;143(8):e254‐e743. 33501848 10.1161/CIR.0000000000000950 PMC13036842

[2] Shah KS , Xu H , Matsouaka RA , et al. Heart failure with preserved, borderline, and reduced ejection fraction: 5‐year outcomes. J Am Coll Cardiol. 2017;70(20):2476‐2486. 29141781 10.1016/j.jacc.2017.08.074

[3] Heidenreich PA , Bozkurt B , Aguilar D , et al. AHA/ACC/HFSA guideline for the management of heart failure: executive summary: a report of the American College of Cardiology/American Heart Association Joint Committee on Clinical Practice Guidelines. J Card Fail. 2022;28(5):810‐830. 10.1016/j.cardfail.2022.02.009 35378259

[4] Solomon SD , McMurray JJV , Anand IS , et al. Angiotensin–neprilysin inhibition in heart failure with preserved ejection fraction. N Engl J Med. 2019;381:1609‐1620. 31475794 10.1056/NEJMoa1908655

[5] McMurray JJV , Solomon SD , Inzucchi SE , et al. Dapagliflozin in patients with heart failure and reduced ejection fraction. N Engl J Med. 2019;381:1995‐2008. 31535829 10.1056/NEJMoa1911303

[6] Armstrong PW , Pieske B , Anstrom KJ , et al. Vericiguat in patients with heart failure and reduced ejection fraction. N Engl J Med. 2020;382(20):1883‐1893. 32222134 10.1056/NEJMoa1915928

[7] Packer M , Anker SD , Butler J , et al. Cardiovascular and renal outcomes with empagliflozin in heart failure. N Engl J Med. 2020;383:1413‐1424. 32865377 10.1056/NEJMoa2022190

[8] Anker SD , Butler J , Filippatos G , et al. Empagliflozin in heart failure with a preserved ejection fraction. N Engl J Med. 2021;385:1451‐1461. 34449189 10.1056/NEJMoa2107038

[9] McMurray JJV , Packer M , Desai AS , et al. Angiotensin‐neprilysin inhibition versus enalapril in heart failure. N Engl J Med. 2014;371(11):993‐1004. 25176015 10.1056/NEJMoa1409077

[10] Greene SJ , Fonarow GC , DeVore AD , et al. Titration of medical therapy for heart failure with reduced ejection fraction. J Am Coll Cardiol. 2019;73(19):2365‐2383. 30844480 10.1016/j.jacc.2019.02.015 PMC7197490

[11] Carnicelli AP , Lippmann SJ , Greene SJ , et al. Sacubitril/Valsartan Initiation and postdischarge adherence among patients hospitalized for heart failure. J Card Fail. 2021;27(8):826‐836. 34364659 10.1016/j.cardfail.2021.03.012

[12] Institute of Medicine . Crossing the Quality Chasm: A New Health System for the 21st Century. The National Academies Press; 2002. 10.17226/10027 25057539

[13] Balas EA , Boren SA . Managing clinical knowledge for health care improvement. Yearb Med Inform. 2000;1:65‐70. 27699347

[14] Ubel PA , Rosenthal MB . Beyond Nudges—when improving health calls for greater assertiveness. N Engl J Med. 2019;380(4):309‐311. 30673544 10.1056/NEJMp1806371

[15] Bello NA , Claggett B , Desai AS , et al. Influence of previous heart failure hospitalization on cardiovascular events in patients with reduced and preserved ejection fraction. Circ Heart Fail. 2014;7(4):590‐595. 24874200 10.1161/CIRCHEARTFAILURE.113.001281 PMC4102617

[16] Rao VN , Murray E , Butler J , et al. In‐hospital initiation of sodium‐glucose cotransporter‐2 inhibitors for heart failure with reduced ejection fraction. J Am Coll Cardiol. 2021;78(20):2004‐2012. 34763778 10.1016/j.jacc.2021.08.064 PMC9766421

[17] Gattis WA , O'connor CM , Gallup DS , Hasselblad V , Gheorghiade M , IMPACT‐HF Investigators and Coordinators . Predischarge initiation of carvedilol in patients hospitalized for decompensated heart failure: results of the initiation management predischarge: process for assessment of carvedilol therapy in heart failure (IMPACT‐HF) trial. J Am Coll Cardiol. 2004;43(9):1534‐1541. 15120808 10.1016/j.jacc.2003.12.040

[18] Velazquez EJ , Morrow DA , DeVore AD , et al. Angiotensin‐neprilysin inhibition in acute decompensated heart failure. N Engl J Med. 2019;380(6):539‐548. 30415601 10.1056/NEJMoa1812851

[19] Morrow DA , Velazquez EJ , DeVore AD , et al. Clinical outcomes in patients with acute decompensated heart failure randomly assigned to sacubitril/valsartan or enalapril in the PIONEER‐HF Trial. Circulation. 2019;139(19):2285‐2288. 30955360 10.1161/CIRCULATIONAHA.118.039331

[20] Bhatt DL , Szarek M , Steg PG , et al. Sotagliflozin in patients with diabetes and recent worsening heart failure. N Engl J Med. 2021;384:117‐128. 33200892 10.1056/NEJMoa2030183

[21] Voors AA , Angermann CE , Teerlink JR , et al. The SGLT2 inhibitor empagliflozin in patients hospitalized for acute heart failure: a multinational randomized trial. Nat Med. 2022;28(3):568‐574. 35228754 10.1038/s41591-021-01659-1 PMC8938265

[22] Clinicaltrials.gov. Dapagliflozin and effect on cardiovascular events in acute heart failure—thrombolysis in myocardial infarction 68 (DAPA ACT HF‐TIMI 68). Accessed May 5, 2022. https://clinicaltrials.gov/ct2/show/NCT04363697.

[23] Chan WV , Pearson TA , Bennett GC , et al. ACC/AHA special report: clinical practice guideline implementation strategies: a summary of systematic reviews by the NHLBI implementation science work group: a report of the American College of Cardiology/American Heart Association Task Force on Clinical Practice Guidelines. J Am Coll Cardiol. 2017;69(8):1076‐1092. 28132746 10.1016/j.jacc.2016.11.004

[24] Ellrodt AG , Fonarow GC , Schwamm LH , et al. Synthesizing lessons learned from Get With The Guidelines: the value of disease‐based registries in improving quality and outcomes. Circulation. 2013;128(22):2447‐2460. 24166574 10.1161/01.cir.0000435779.48007.5c

[25] Get with the guidelines heart failure . Accessed April 15, 2022. https://www.heart.org/en/professional/quality-improvement/get-with-the-guidelines/get-with-the-guidelines-heart-failure

[26] Bahiru E , Ziaeian B , Moucheraud C , et al. Association of dual eligibility for Medicare and Medicaide with heart failure quality and outcomes among get with the guidelines‐heart failure hospitals. JAMA Cardiol. 2021;6(7):791‐800. 33825802 10.1001/jamacardio.2021.0611 PMC8027938

[27] Heidenreich PA , Hernandez AF , Yancy CW , Liang L , Peterson ED , Fonarow GC . Get with the guidelines program participation, process of care, and outcome for Medicare patients hospitalized with heart failure. Circ Cardiovasc Qual Outcomes. 2012;5(1):37‐43. 22235067 10.1161/CIRCOUTCOMES.110.959122

[28] DeVore AD , Cox M , Heidenreich PA , et al. Cluster‐randomized trial of personalized site performance feedback in get with the guidelines‐heart failure. Circ Cardiovasc Qual Outcomes. 2015;8(4):421‐427. 26175533 10.1161/CIRCOUTCOMES.114.001333

[29] DeVore AD , Granger BB , Fonarow GC , et al. Care optimization through patient and hospital engagement clinical trial for heart failure: rationale and design of CONNECT‐HF. Am Heart J. 2020;220:41‐50. 31770656 10.1016/j.ahj.2019.09.012

[30] DeVore AD , Granger BB , Fonarow GC , et al. Effect of a hospital and postdischarge quality improvement intervention on clinical outcomes and quality of care for patients with heart failure with reduced ejection fraction: the CONNECT‐HF randomized clinical trial. JAMA. 2021;326(4):314‐323. 34313687 10.1001/jama.2021.8844 PMC8317015

[31] Desai AS , Maclean T , Blood AJ , et al. Remote optimization of guideline‐directed medical therapy in patients with heart failure with reduced ejection fraction. JAMA Cardiol. 2020;5(12):1430‐1434. 32936209 10.1001/jamacardio.2020.3757 PMC7495335

[32] Ghazi L , Yamamoto Y , Riello RJ , et al. Electronic alerts to improve heart failure therapy in outpatient practice: a cluster randomized trial. J Am Coll Cardiol . Forthcoming 2022. https://www.jacc.org/doi/10.1016/j.jacc.2022.03.338 10.1016/j.jacc.2022.03.338 35385798

[33] Allen LA , Venechuk G , McIlvennan CK , et al. An electronically delivered patient‐activation tool for intensification of medications for chronic heart failure with reduced ejection fraction: the EPIC‐HF trial. Circulation. 2021;143(5):427‐437. 33201741 10.1161/CIRCULATIONAHA.120.051863 PMC7855616

[34] DeVore AD , Wosik J , Hernandez AF . The future of wearables in heart failure patients. JACC Heart Fail. 2019;7(11):922‐932. 31672308 10.1016/j.jchf.2019.08.008

[35] Clinicaltrials.gov. Artificial intelligence mobile health trial of a digital platform to optimize GDMT using wearable sensors (AIM‐POWER). Accessed April 15, 2022. https://clinicaltrials.gov/ct2/show/NCT04191330

[36] Heidenreich PA , Bozkurtchair B , Aguilar D , et al. AHA/ACC/HFSA guideline for the management of heart failure: executive summary: a report of the American College of Cardiology/American Heart Association Joint Committee on Clinical Practice Guidelines. J Card Fail. 2022:​ S1071‐S9164. 10.1016/j.cardfail.2022.02.009 35379504

[37] Sarkies MN , Jones LK , Gidding SS , Watts GF . Improving clinical practice guidelines with implementation science. Nat Rev Cardiol. 2022;19(1):3‐4. 34799708 10.1038/s41569-021-00645-x

[38] Pandey A , Keshvani N , Zhong L , et al. Temporal trends and factors associated with cardiac rehabilitation participation among Medicare beneficiaries with heart failure. JACC Heart Fail. 2021;9(7):471‐481. 33992563 10.1016/j.jchf.2021.02.006

[39] Report to the Congress: Medicare and the health care delivery system—mandated report: the effects of the Hospital Readmissions Reduction Program. Washington , DC: Medicare Payment Advisory Commission, 2018. https://www.medpac.gov/document-type/report/

[40] Wadhera RK , Joynt Maddox KE , Wasfy JH , Haneuse S , Shen C , Yeh RW . Association of the hospital readmissions reduction program with mortality among Medicare beneficiaries hospitalized for heart failure, acute myocardial infarction, and pneumonia. JAMA. 2018;320(24):2542‐2552. 30575880 10.1001/jama.2018.19232 PMC6583517

[41] Jeffery RA , To MJ , Hayduk‐Costa G , et al. Interventions to improve adherence to cardiovascular disease guidelines: a systematic review. BMC Fam Pract. 2015;16:147. 26494597 10.1186/s12875-015-0341-7 PMC4619086

[42] Samsky MD , Lin L , Greene SJ , et al. Patient perceptions and familiarity with medical therapy for heart failure. JAMA Cardiol. 2020;5(3):292‐299. 31734700 10.1001/jamacardio.2019.4987 PMC6865328

[43] Greene SJ , DeVore AD , Sheng S , et al. Representativeness of a heart failure trial by race and sex: results from ASCEND‐HF and GWTG‐HF. JACC Heart Fail. 2019;7(11):980‐992. 31606362 10.1016/j.jchf.2019.07.011 PMC7362664

[44] Fudim M , Sayeed S , Xu H , et al. Representativeness of the PIONEER‐HF clinical trial population in patients hospitalized with heart failure and reduced ejection fraction. Circ Heart Fail. 2020;13(4):e006645. 32248695 10.1161/CIRCHEARTFAILURE.119.006645 PMC8819257

[45] O'connor KD , Brophy T , Fonarow GC , et al. Testing for coronary artery disease in older patients with new‐onset heart failure: findings from get with the guidelines‐heart failure. Circ Heart Fail. 2020;13(4):006963. 10.1161/CIRCHEARTFAILURE.120.006963 PMC10790075 32207996

[46] Zheng J , Heidenreich PA , Kohsaka S , Fearon WF , Sandhu AT . Variability in coronary artery disease testing for patients with new‐onset heart failure. J Am Coll Cardiol, 79(9):849‐860. 35241218 10.1016/j.jacc.2021.11.061 PMC9031351

